# Co-expressed genes enhance precision of receptor status identification in breast cancer patients

**DOI:** 10.1007/s10549-018-4920-x

**Published:** 2018-08-16

**Authors:** Michael Kenn, Dan Cacsire Castillo-Tong, Christian F. Singer, Michael Cibena, Heinz Kölbl, Wolfgang Schreiner

**Affiliations:** 1 Section of Biosimulation and Bioinformatics, Center for Medical Statistics, Informatics and Intelligent Systems (CeMSIIS), Medical University of Vienna, Spitalgasse 23, 1090 Vienna, Austria; 2 Translational Gynecology Group, Department of Obstetrics and Gynecology, Comprehensive Cancer Center, Medical University of Vienna, Waehringer Guertel 18-20, 1090 Vienna, Austria; 3 Department of General Gynecology and Gynecologic Oncology, and Comprehensive Cancer Center, Medical University of Vienna, Waehringer Guertel 18-20, 1090 Vienna, Austria

**Keywords:** Gene expression, Breast cancer, Receptor status, Precision medicine, Data science, Mathematical oncology

## Abstract

**Purpose:**

Therapeutic decisions in breast cancer patients crucially depend on the status of estrogen receptor, progesterone receptor and HER2, obtained by immunohistochemistry (IHC). These are known to be inaccurate sometimes, and we demonstrate how to use gene-expression to increase precision of receptor status.

**Methods:**

We downloaded data from 3241 breast cancer patients out of 36 clinical studies. For each receptor, we modelled the mRNA expression of the receptor gene and a co-gene by logistic regression. For each patient, predictions from logistic regression were merged with information from IHC on a probabilistic basis to arrive at a fused prediction result.

**Results:**

We introduce Sankey diagrams to visualize the step by step increase of precision as information is added from gene expression: IHC-estimates are qualified as ‘confirmed’, ‘rejected’ or ‘corrected’. Additionally, we introduce the category ‘inconclusive’ to spot those patients in need for additional assessments so as to increase diagnostic precision and safety.

**Conclusions:**

We demonstrate a sound mathematical basis for the fusion of information, even if partly contradictive. The concept is extendable to more than three sources of information, as particularly important for OMICS data. The overall number of undecidable cases is reduced as well as those assessed falsely. We outline how decision rules may be extended to also weigh consequences, being different in severity for false-positive and false-negative assessments, respectively. The possible benefit is demonstrated by comparing the disease free survival between patients whose IHC could be confirmed versus those for which it was corrected.

**Electronic supplementary material:**

The online version of this article (10.1007/s10549-018-4920-x) contains supplementary material, which is available to authorized users.

## Introduction

### Background and significance

Individualized breast cancer therapy is based on molecular characterization [1–3], in particular the presence of receptors for estrogen (ER), progesterone (PGR) and human epidermal growth factor 2 (HER2) in an incoming patient. It is hence essential to reliably assess the status of these three receptors when aiming at optimum individualized therapy within precision medicine [1–5].

Receptor status obtained from immunohistochemistry (IHC) is usually considered standard of care, and crucially guides therapy. However, in up to 20% of patients, assigned ER^+^ status may be erroneously classified [6–8]. Multicenter studies have been performed for quality assessment [9, 10] and guidelines have been issued [8, 11]. Possible consequences of misclassification on outcome have been evaluated [12] and several authors have suggested making improvements on the reliability of IHC estimates by additionally considering gene-expression data [13–16].

In a previous paper [17], we have substantiated this suggestion by devising refined decision criteria based on gene-expression data.

### Receptor status from IHC and one single gene

Our previous work [17] started out from IHC measurements (e.g. ${\text{ER}}_{{{\text{IHC}}}}^{+}$, ${\text{ER}}_{{{\text{IHC}}}}^{ - }$ and ${\text{ER}}_{{{\text{IHC}}}}^{0}$ for estrogen positive, negative or missing). In a second step, estimates for gene-expression (GE) were added for ER (gene *ESR1*), for PGR (gene *PGR*) and for HER2 (gene *ERBB2*). Combined results were obtained in each patient via a scoring system based on all three receptors.

As a result, the IHC estimates of receptor status were questioned in a significant portion of patients. These patients might receive more adequate treatment due to an improvement of receptor status assessment, as proposed.

### Adding co-genes

In the present work we now extend our previous analysis to qualified co-genes as suggested by several authors [18, 19]. We were able to demonstrate how adding co-expression (CO) can even further improve receptor status assessment.

**Fig. 1:**
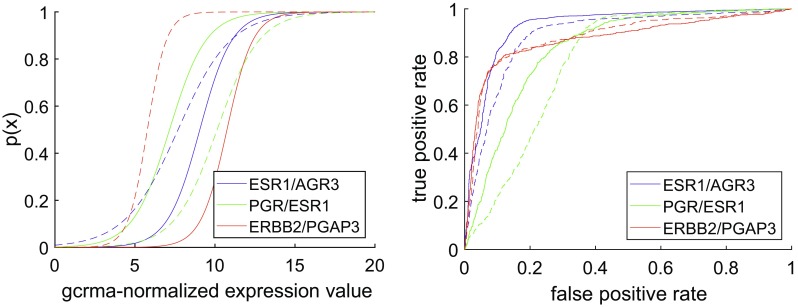
Logistic regressions of IHC-obtained receptor status versus gene expression. For each receptor we obtain one curve from the very receptor gene (solid curve) and a second one from the co-gene (dashed), both shown in the same colour (see legend). Left panel: probabilities (*y*-axis) of positive receptor status, given a GCRMA-normalized expression value (*x*-axis). For values of regression parameters and quality of regression see Table 8. Right panel: corresponding receiver operator characteristics (ROC)-curves. For quantitative estimates of regression quality, see Table 8

We first demonstrate how co-genes can be properly selected and why we ultimately chose *AGR3* as co-gene for *ESR1* [20, 21], *ESR1* as co-gene for PGR and *PGAP3* as co-gene for HER2, see Fig. 1 and the “Results”. For probe sets and statistical parameters see supplementary material.

### Objectives regarding patient benefit

The usefulness of our method is assessed as follows:

- Disease free survival curves are compared for those patients having their IHC estimate confirmed by both, GE as well as CO. They have received optimum therapy, as concluded from IHC alone. Second, we compute the disease free survival for those patients whose IHC estimates have been questioned by GE and/or CO. Therapies might have been erroneous, or at least suboptimal. The difference in disease free survival is considered a direct indicator of a benefit possibly being leveraged by this work.
- Paired survival curves are computed for the ER, PGR and HER2.

## Results

### Predictive co-genes

All genes were subjected to a numerical ‘co-expression check’ to ascertain their usability, for details see the methods section. All in all we ended up with pairs of receptor-genes and co-genes as shown in Table 1.

**Table 1:** Receptor-genes and co-genes

Receptor	Receptor gene	Co-gene
Estrogen receptor (ER)	*ESR1*	*AGR3*
Progesterone receptor (PGR)	*PGR*	*ESR1*
Human epidermal growth factor receptor 2 (HER2)	*ERBB2*	*PGAP3*

### Predicting receptor status separately from genes and co-genes

For a given receptor, such as the ER, we performed two separate logistic regressions, one for the very receptor gene and a second one for a co-gene, see Fig. 1, left panel.

Each curve is represented by a logit function. For simplicity of notation, we exemplify the formalism only for the estrogen receptor:

1 $${p_{{\text{ER}}_{{{\text{GE}}}}^{+}}}({x_{{\text{GE}}}})\,=\,\frac{1}{{1+{e^{\beta _{0}^{{{\text{GE}}}}+\,\beta _{1}^{{{\text{GE}}}}\,{x_{{\text{GE}}}}}}}}$$

The differences between the curves in Fig. 1 are reflected in individual parameters *β*_0_ and *β*_1_, resulting from different logistic regressions for each gene and co-gene. See supplementary material for numerical results and the methods section for computational details.

Upon entering the expression value, *x*_GE_, above formula yields the probability ${p_{{\text{ER}}_{{{\text{GE}}}}^{+}}}({x_{{\text{GE}}}})$ for the patient being receptor positive. Vice versus, the probability for being receptor negative is given by ${p_{{\text{ER}}_{{{\text{GE}}}}^{ - }}}({x_{{\text{GE}}}})\,=\,1 - {p_{{\text{ER}}_{{{\text{GE}}}}^{+}}}({x_{{\text{GE}}}})$.

A similar formula is obtained for the co-gene of estrogen, *AGR3*, with different coefficients *β*_0_ and *β*_1_, however. Thus, for a given receptor being positive we end up with two probabilities, ${p_{{\text{ER}}_{{{\text{GE}}}}^{+}}}({x_{{\text{GE}}}})$ and ${p_{{\text{ER}}_{{{\text{CO}}}}^{+}}}({x_{{\text{CO}}}})$.

The very same procedure applies to PGR and HER2. Mathematical details and values for *β*_0_ and *β*_1_ are given in supplementary material. Note that all curves tend towards *p*(*x*) = 1, since very high expression indicates receptor positivity with almost certainty.

### Joint prediction of receptor status from IHC, genes and co-genes

In this section we demonstrate the benefit achieved by enriching IHC estimates with information from receptor-genes and co-genes.

Considering only IHC estimates, numbers of patients are given in column ‘IHC only’ of Table 2. Results ‘−’ and ‘+’ directly enter treatment allocation, patients with IHC estimates ‘not available’ cannot be properly allocated (no conclusions can be drawn, hence we use the term ‘inconclusive’ for the rest of this article).

**Table 2:**
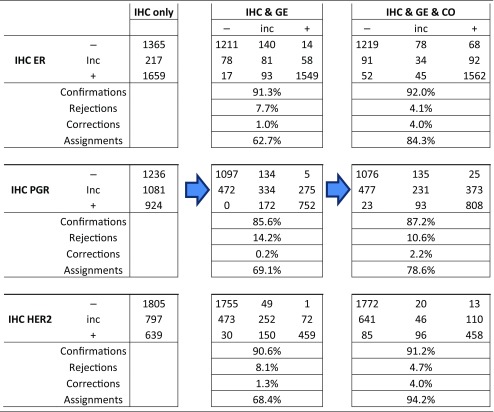
Results of joint prediction from IHC, genes and co-genes

Results are given separately for each receptor. For IHC (leftmost column) we discern the categories—/inconclusive (inc)/ +. In some cases information from IHC is not available but we use the term ‘inconclusive’ for consistency of notation. Information from gene expression (GE, CO) is but always available, however it may yield ‘inconclusive’ as a result, see the column headings

#### Probabilistic view on IHC estimates

As a first step towards joining information from IHC and gene-expression (Fig. 1), IHC estimates are interpreted probabilistically as follows:

1. If an IHC-assay yields receptor positive, we do not take this for sure but attribute the precision $p_{{{\text{IHC}}}}^{+}=0.85$ for the sample being truly positive and insert this into Eq. 2. This is reasonable, since we have to bear in mind that about 15% of IHC estimates are considered false [6, 7].
2. Conversely, if an IHC-assay yields receptor negative, we credit $p_{{{\text{IHC}}}}^{+}=0.15$ for truly being receptor positive.
3. If an IHC estimate is not available, we attribute the precision of $p_{{{\text{IHC}}}}^{+}=0.5$. Note that this precision bears no context to the prevalence of receptor status.

#### Joint prediction from IHC, expression of genes and co-genes

For a specific patient, the probabilities obtained from IHC, gene-expression and co-expression have now to be fused to arrive at a joint estimate.

For reasons outlined in the methods section, we consider odds, aggregate them by adding their logarithms and arrive at a score *S*^+^ for the patient being receptor positive:

2 $${S^+}\,=\begin{array}{*{20}{c}} {\log \left( {\frac{{p_{{{\text{IHC}}}}^{+}}}{{1 - p_{{{\text{IHC}}}}^{+}}}} \right)}&{ - (\beta _{0}^{{{\text{GE}}}}+\beta _{1}^{{{\text{GE}}}}{x_{{\text{GE}}}})}&{ - (\beta _{0}^{{{\text{CO}}}}+\beta _{1}^{{{\text{CO}}}}{x_{{\text{CO}}}})} \\ {\log \,{\text{odds}}\,{\text{IHC}}}&{\log \,{\text{odds}}\,{\text{GE}}}&{{\text{log}}\,{\text{odds}}\,{\text{CO}}} \end{array}$$

Numerical values for the parameters *β* are given in supplementary material, for each of the receptors. To arrive at a decision, this score *S*^+^ is compared with a threshold, *S*_0_, which we set ${S_0}\,=\,\log (0.85/0.15)\, \approx \,1.735\,=\,{\text{logit(precision)}}$.^1^ This represents an executable procedure for aggregating information into a comprehensive receptor status assessment:

3 $$\begin{gathered} {\text{if}}\,{S^+}\,>\,{S_0} \to \,{\text{receptor}}\,{\text{positive}} \hfill \\ {\text{if}}\,{S^+}\,<\, - {S_0} \to \,{\text{receptor}}\,{\text{negative}} \hfill \\ {\text{if}}\, - {S_0}\, \le \,{S^+}\, \le \,{S_0} \to \,{\text{inconclusive}} \hfill \\ \end{gathered}$$

For mathematical details and threshold setting, please see the methods section.

Combining information from IHC, gene-expression and co-expression yields the numbers of patients as shown in the rightmost parts of Table 2, columns ‘IHC & Ge & CO’.

### Overall improvement of receptor diagnostics based on joint assessment

**Fig. 2:**
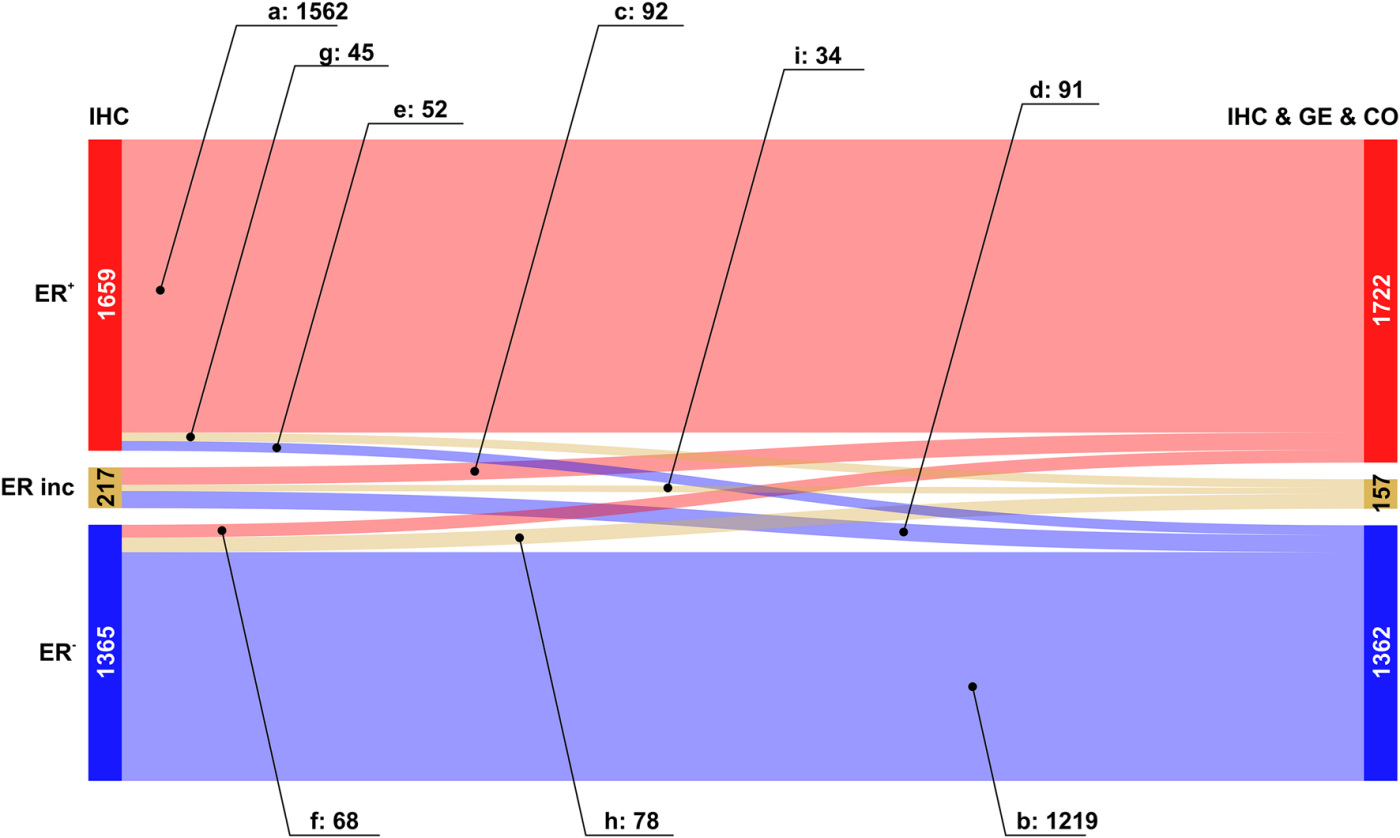
Overall improvement of estrogen receptor assessment. Colour code for categories of receptor assessment: red: receptor positive (+), beige: receptor status inconclusive, blue: receptor negative (−). Note that the category ‘inconclusive’ for IHC in fact means that the IHC estimate is missing. Left sidebar of Sankey diagram: number of patients classified on basis of ‘IHC only’ (red: ER^+^, beige: ER^inc^, blue: ER^−^). Right sidebar of Sankey diagram: number of patients classified when considering joint information from IHC, expression of the receptor gene GE, (*ESR1*) and the co-gene, CO, (*AGR3*). Flows from left (‘IHC only’) to right (IHC & GE & CO) are coloured according to their final category. Numbers of patients are given together with labels of flows (a–i)

**Fig. 3:**
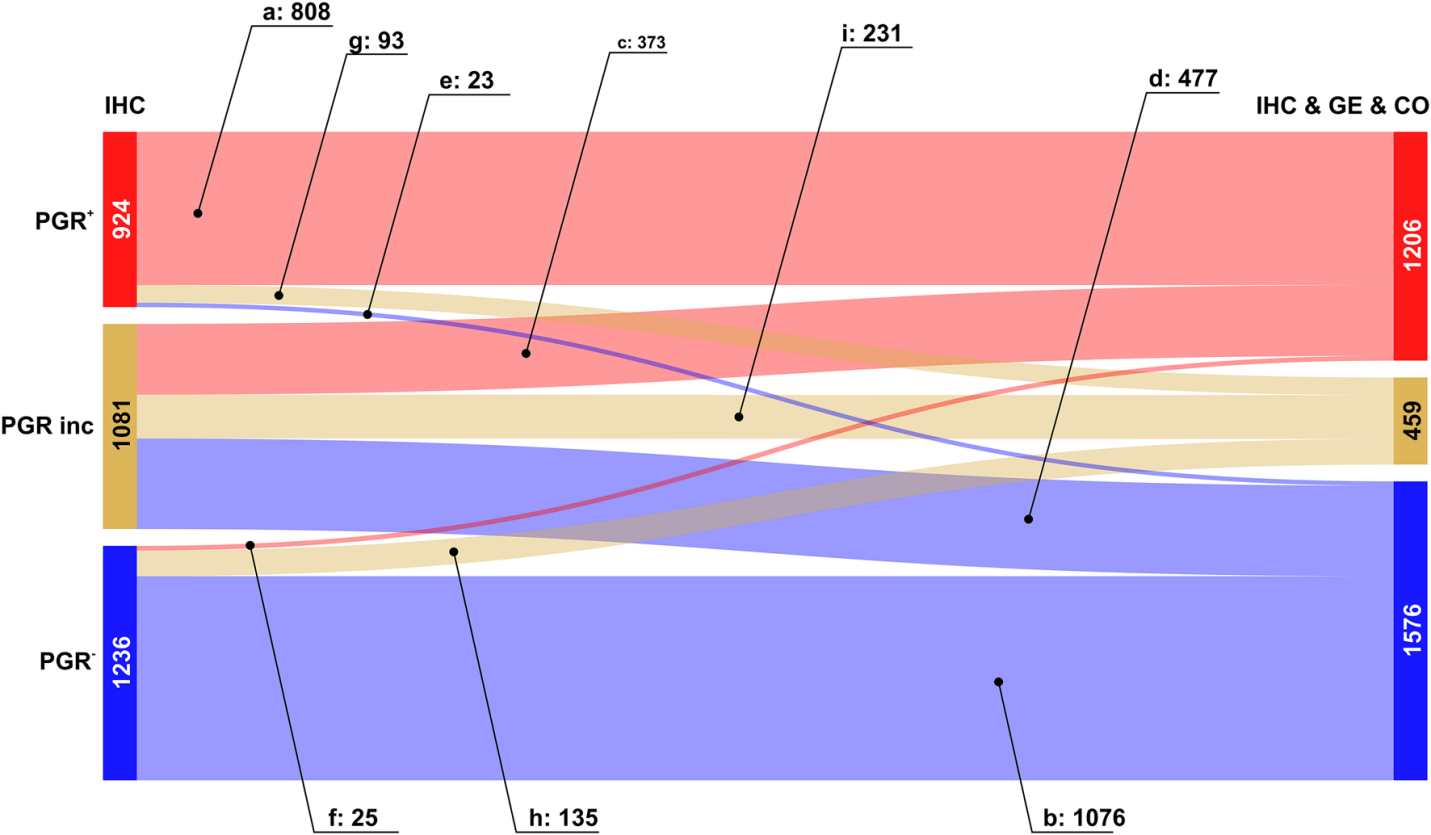
Overall improvement of progesterone receptor assessment. Colour code for categories of receptor assessment: red: receptor positive (+), beige: receptor status inconclusive, blue: receptor negative (−). Note that the category ‘inconclusive’ for IHC in fact means that the IHC estimate is missing. Left sidebar of Sankey diagram: number of patients classified on basis of ‘IHC only’ (red: PGR^+^, beige: PGR^inc^, blue: PGR^−^). Right sidebar of Sankey diagram: number of patients classified when considering joint information from IHC, expression of the receptor gene GE, (*PGR*) and the co-gene, CO, (*ESR1*). Flows from left (‘IHC only’) to right (IHC & GE & CO) are coloured according to the category assigned under full information (IHC & GE & CO)

**Fig. 4:**
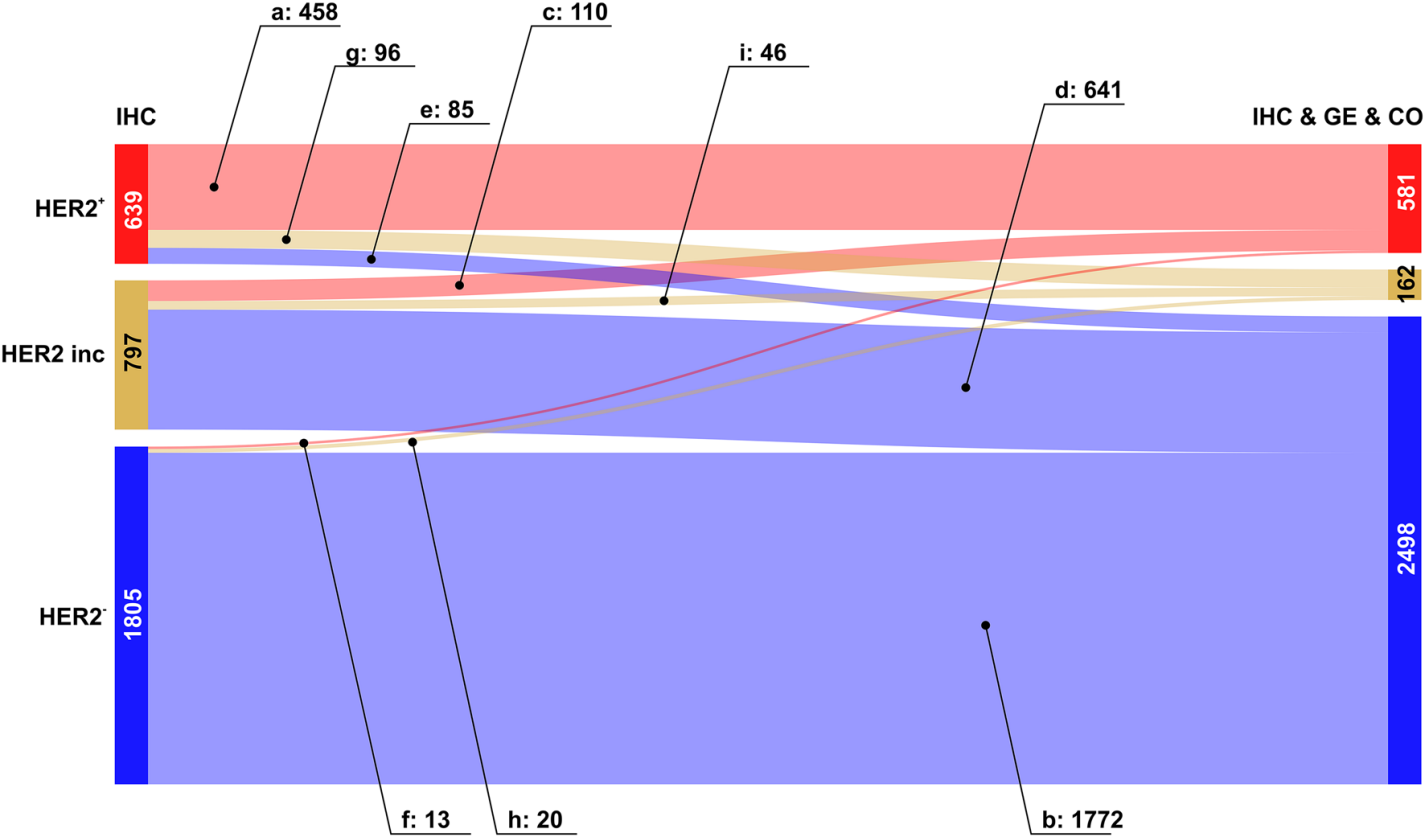
Overall improvement of HER2 assessment. Colour code for categories of receptor assessment: red: receptor positive (+), beige: receptor status inconclusive, blue: receptor negative (−). Note that the category ‘inconclusive’ for IHC in fact means that the IHC estimate is missing. Left sidebar of Sankey diagram: number of patients classified on basis of ‘IHC only’ (red: HER2^+^, beige: HER2^inc^, blue: HER2^−^). Right sidebar of Sankey diagram: number of patients classified when considering joint information from IHC, expression of the receptor gene GE, (*ERBB2*) and the co-gene, CO, (*PAGP3*). Flows from left (‘IHC only’) to right (IHC & GE & CO) are coloured according to their final category

We then analysed the overall improvement of receptor assessment due to adding expression data for the receptor gene and a co-gene. To illustrate the overall effect of such a joint assessment, flows of patients between diagnostic states ‘IHC’ and ‘IHC & GE & Co’ are shown in a Sankey diagram, see Figs. 2, 3 and 4.

The Sankey diagram displays changes in estimated receptor status (‘flows’ of patients) after enriching information from IHC by information from GE and CO.

Since we discriminate three different categories (‘+’, ‘−’ and ‘inconclusive’), there are 9 possible types of flow from initial IHC estimates towards some final result which is based on all information available (IHC & GE & CO). Flows are labelled from (a) to (i), see also Table 3, and the examples below for ER, PGR and HER2.

**Table 3:** Flows of patients due to refined receptor diagnosis

Flow-label	Flow-category	IHC category		IHC & GE & CO category
(a)	Confirmed +	Definite +	→	Definite +
(b)	Confirmed −	Definite −	→	Definite −
(c)	Allocated +	Inconclusive	→	Definite +
(d)	Allocated −	Inconclusive	→	Definite −
(e)	Corrected to −	Definite +	→	Definite −
(f)	Corrected to +	Definite −	→	Definite +
(g)	Rejected +	Definite +	→	Inconclusive
(h)	Rejected −	Definite −	→	Inconclusive
(i)	Undetermined	Inconclusive	→	Inconclusive

Labels (a–i) are used in text and figures to reference specific flows. Each flow represents the change in category (definite −, definite +, inconclusive) due to enriched information

The relevance of this sort of enriched receptor diagnosis is reflected in the fact that out of 9 patient flows possible in theory, each one actually occurs in practice.

#### Estrogen receptor assessment

As expected, the flow category ‘*confirmed’* of the IHC estimates represent the largest flows [in Fig. 2: red → red (label a: 1562, ≈ 94%) and blue → blue (label b: 1219, ≈ 89%)]. The error rates reported (6% and 11%, respectively) are only seemingly contradictive with the initial guess of 15%, in fact they are not. 15% invalid IHC results have been reported in the literature (as quoted). Adding gene plus co-gene information fixes only a portion—not all of those.

Very important are flows *allocating* missing IHC estimates from ‘inconclusive’ into ‘definite’, after adding information from GE & CO. They represent diagnostic improvements, resulting in ER^+^ for ≈ 42% (92 patients) and in ER^−^ for ≈ 42% (91 patients), see Fig. 2, labels (c) and (d), respectively.

Of utmost interest for patient safety are *‘corrected’* cases, in which the IHC estimate is converted into its opposite. Fortunately, we found only a few such cases: 52 (≈ 3%) correcting ER^+^ → ER^−^ and 68 (≈ 5%) correcting ER^−^ → ER^+^, see labels (e) and (f), respectively. Even though improvements are small in terms of percentages, they helped to fine tune the treatment approach and be more precise in treatment selection for better results.

A third type of flow represents *‘rejected’* estimates, i.e. patients starting with a definite IHC estimate, which is questioned thereafter and ends up inconclusive after adding ‘GE & CO’. In our data we observe 45 such cases for ER^+^ (≈ 3%) and 78 for ER^−^ (≈ 6%), see Fig. 2, labels (g) and (h), respectively. These cases also represent an improvement, even though the receptor status results inconclusive and has to be re-determined: This way, possible suboptimal treatments may be avoided.

The last flow represents ‘*inconclusive*’ patients (in our data 34, i.e. ≈ 16%) for which not even the full information (IHC & GE & CO) sufficed to arrive at a definite receptor status, see Fig. 2, label (i).

The overall improvement of estrogen receptor diagnostics due to our proposed procedure is reflected in the increase of definite results by ≈ 2%, from 3024 (= 1659 + 1365) to 3084 (= 1722 + 1362), cf. Table 2 and Fig. 2. Concordantly, the number of receptor inconclusive declines from 217 to 157, i.e. to ≈ 28%.

#### Progesterone receptor assessment

In most cases, enhanced information leads to the confirmation of PGR-status, see Fig. 3: red → red (label a: 808 patients) and blue → blue (label b: 1076 patients).

IHC estimates initially missing were upgraded into definitely PGR^+^ in a flow comprising 373 patients and into definite PGR^−^ in 477 patients, see Fig. 3, labels (c) and (d), respectively.

Cases in which PGR-status needs to be corrected are rare: 23 turning PGR^+^→PGR^−^ (label e) and 25 PGR^−^→ PGR^+^ (label f), see the faint blue and red ribbons crossing over into the opposite zone.

The flows leading into assessments in question are moderate in size: 93 patients initially within PGR^+^ evade to ‘inconclusive’, see Fig. 3, label (g), and 135 initially PGR^−^ end up ‘inconclusive’, see Fig. 3, label (h). As mentioned above for ER status, the category ‘inconclusive’ being rendered may be seen as a warning to improve assessment (in which way ever) so as to avoid possibly suboptimal treatment.

Inconclusive PGR-status remains as such in 231 patients, despite full information, see Fig. 3, label (i).

The overall improvement of PGR diagnostics is reflected in the increase of definite results from 2160 (= 924 + 1236) to 2882 (= 1206 + 1576), cf. Table 2 and Fig. 3. Concomitantly, the number of inconclusive receptor estimates declines from 1081 to 459.

#### HER2 assessment

Despite the availability of standardized HER2 testing strategies and the widespread use of ASCO/CAP guidelines, amplification results vary considerably. Our approach to enrich information for HER2 assessment, leads to confirmation in about 72% of ${\text{HER2}}_{{{\text{IHC}}}}^{+}$ patients, see Fig. 4, flow labelled a: 458 patients out of 639. For ${\text{HER2}}_{{{\text{IHC}}}}^{ - }$ even the vast majority of estimates is confirmed: flow labelled b: 1772 out of 1805.

The flow turning missing IHC estimates (${\text{HER2}}_{{{\text{IHC}}}}^{{{\text{inc}}}}$) into definitely HER2^+^ comprises 110 patients (out of 797), which is about 14%. About 80% (641) turn into HER2^−^ see Fig. 4, labels (c) and (d), respectively.

Corrected cases for HER2 are asymmetric: 85 turn HER2^+^→ HER2^−^ (≈ 13%, label e) and 13 HER2^−^→ HER2^+^ (≈ 1%, label f), see the blue and the faint red flow crossing over into the opposite domains, respectively.

Flows representing questioned assessments have considerable magnitude for patients initially diagnosed HER2^+^: 96 patients (≈ 15%) evade to ‘inconclusive’, see Fig. 4, flow labelled (g). Conversely, only 20 (≈ 1%) of those initially diagnosed HER2^−^ are questioned and end up ‘inconclusive’, see Fig. 3, flow labelled (h). As mentioned above, questioned estimates offer the chance to avoid possibly suboptimal treatments.

Inconclusive HER2-status in 797 patients remains inconclusive in 46 patients (≈ 6%), see Fig. 4, flow labelled (i).

The overall improvement of HER2 diagnostics is reflected in the increase of definite results by ≈ 26%, from 2444 (= 639 + 1805) to 3079 (= 581 + 2498), cf. Table 2 and Fig. 4. Concordantly, the number of receptor inconclusive declines from 797 to 162 (decline to ≈ 20%).

## Discussion

### Selection of co-genes

One would expect co-genes could be found by looking for genes which show the strongest correlation with the corresponding receptor gene. This is not optimum, however, for the following reason: Given a gene with 100% correlation, it could clearly deliver no additional information on top of the gene itself. Hence, looking for largest possible correlations is suboptimal.

For this reason we applied linear discriminant analysis via the limma software package, as described in the methods section, results for the estrogen receptor see table 4. Discriminant analysis in fact led to the surprising finding that a co-gene (in this case ERS1) of progesterone may be more predictive than the very receptor gene itself (*PGR*).

**Table 4:** Probe sets allowing for classification of estrogen receptor (ER) status

Rank	Gene	Probe set	*t-*value
1	*ESR1*	205225_at	75.2026
2	*AGR3*	228241_at	64.9077
3	*CA12*	204508_s_at	60.0012
4	*CA12*	214164_x_at	58.8398
5	*CA12*	215867_x_at	58.3216
6	*CA12*	203963_at	56.0489
7	*TBC1D9*	212956_at	55.7256
8	*PSAT1*	223062_s_at	55.4939
9	*GATA3*	209603_at	55.0988
10	*GATA3*	209602_s_at	53.5509

The top 10 probe sets list is sorted by descending *t*-values. *ESR1* is the receptor gene itself, ‘estrogen receptor 1’, scoring highest. The second, *AGR3* is taken as co-gene. Note that sorting according to ascending *p*-values would entail the very same ranking. However, *p*-values result exceedingly small due to the very large number of samples, and their values are hence meaningless in the present context. Hence we refrain from listing them. The same holds for Tables 6 and 7

### Concordance of estrogen and progesterone receptor status

ER and PGR are concordant in the majority of cases. However, in accordance with literature [8] a small portion (23 ≈ 1.7%) of the patients assessed ${\text{ER}}_{{{\text{IHC}}}}^{ - }$ were at the same time found ${\text{PGR}}_{{{\text{IHC}}}}^{+}$ in our dataset, see Table 5. Likewise, 240 patients assessed ${\text{PGR}}_{{{\text{IHC}}}}^{ - }$ were at the same time found ${\text{ER}}_{{{\text{IHC}}}}^{+}$.

**Table 5:** Concordance of IHC estimates for estrogen and progesteron

	${\text{PGR}}_{{{\text{IHC}}}}^{+}$	${\text{PGR}}_{{{\text{IHC}}}}^{{{\text{inc}}}}$	${\text{PGR}}_{{{\text{IHC}}}}^{ - }$
${\text{ER}}_{{{\text{IHC}}}}^{+}$	901	518	240
${\text{ER}}_{{{\text{IHC}}}}^{{{\text{inc}}}}$	0	216	1
${\text{ER}}_{{{\text{IHC}}}}^{ - }$	23	347	995

As a consequence, both receptors have to be considered in combination to optimize the stratification of therapies.

**Table 6:** Probe sets allowing for classification of progesterone receptor (PGR) status

Rank	Gene	Probe set	*t-*value
1	*PGR*	228554_at	50.9031
2	*ESR1*	205225_at	43.0697
3	*AGR3*	228241_at	41.2904
4	*CA12*	204508_s_at	40.7144
5	*CA12*	214164_x_at	39.7163
6	*CA12*	215867_x_at	39.3184
7	*CA12*	203963_at	38.6599
8	*GREB1*	205862_at	38.5008
9	*SCUBE2*	219197_s_at	38.2929
10	*GFRA1*	230163_at	37.2852

The list is sorted by descending *t*-values. *PGR* is the receptor gene itself, scoring highest. Remarkably, *ESR1*, the very receptor gene for estrogen, scores second highest. Nevertheless we take it as co-gene for *PGR*

**Table 7:** Probe sets allowing for classification of HER2 status

Rank	Gene	Probe set	*t-*value
1	*PGAP3*	55616_at	56.6386
2	*ERBB2*	234354_x_at	55.7404
3	*PGAP3*	221811_at	54.9610
4	*MIEN1*	224447_s_at	52.7986
5	*STARD3*	202991_at	47.7318
6	*ERBB2*	216836_s_at	44.4821
7	*GRB7*	210761_s_at	40.9352
8	*ERBB2*	210930_s_at	33.7941
9	*ORMDL3*	223259_at	32.7630
10	*CDK12*	225691_at	32.2625

The list is sorted by descending *t*-values. *ERBB2* is the receptor gene itself, scoring second. Highest scores *PGAP3*, taken as co-gene

### Impact of false positive hormone receptor assessment on outcome

In clinical practice, therapy is allocated according to IHC estimates. We know, however, that these may sometimes be inaccurate, and we have to envisage worse outcomes as compared to patients with correctly assessed receptor status. In order to quantify these effects (based on our model with parameters given in Table 8) we build sets of patients as follows, cf. Fig. 2:

**Table 8:** Receptor-genes, co-genes and parameters from logistic regression

		Probe set	Logistic regression parameters		Logistic regression quality		
			$\beta _{0}^{{{\text{GE}}}}$	$\beta _{1}^{{{\text{GE}}}}$	AUC	Dev of fit DoF	No. of samples
ER							
Gene	*ESR1*	205225_at	8.98	− 0.99	0.95	1654.9	3024
Co-gene	*AGR3*	228241_at	4.64	− 0.60	0.92	2071.4	
PGR							
Gene	*PGR*	228554_at	6.25	− 0.87	0.92	1522.1	2160
Co-gene	*ESR1*	205225_at	7.67	− 0.76	0.88	1715.1	
HER2							
Gene	*ERBB2*	216836_s_at	13.20	− 1.23	0.90	1491.4	2444
Co-gene	*PGAP3*	221811_at	9.69	− 1.68	0.91	1374.1	

Probe sets refer to the Affymetrix chip U133A + 2.0. AUC means ‘area under the curve’ and DoF means ‘deviance of fit’, see page 118 in [22]. For the regression coefficients *β*_i_ we show *p*-values for being non-zero

1. The set {ER_a_} of patients assessed estrogen positive by IHC and being confirmed by GE & CO, labelled flow a in Fig. 2 and comprised of 1562 patients. We may assume that they received anti-hormone therapy, as was adequate for them.
2. The set {ER_e_} of patients assessed ER positive by IHC but being corrected by GE & CO, see flow e, 52 patients.
3. The set {ER_g_} of patients assessed ER positive by IHC but rejected by GE & CO, see flow g, 45 patients.
4. The merger set {ER_e,g_} = {ER_e_} ∪ {ER_g_} of patients assessed ER positive by IHC but either corrected or rejected by GE & CO, 97 patients. We may assume that these patients have received anti-hormone therapy which might have been ineffective. At the same time they were deprived of necessary chemotherapy.

**Fig. 5:**
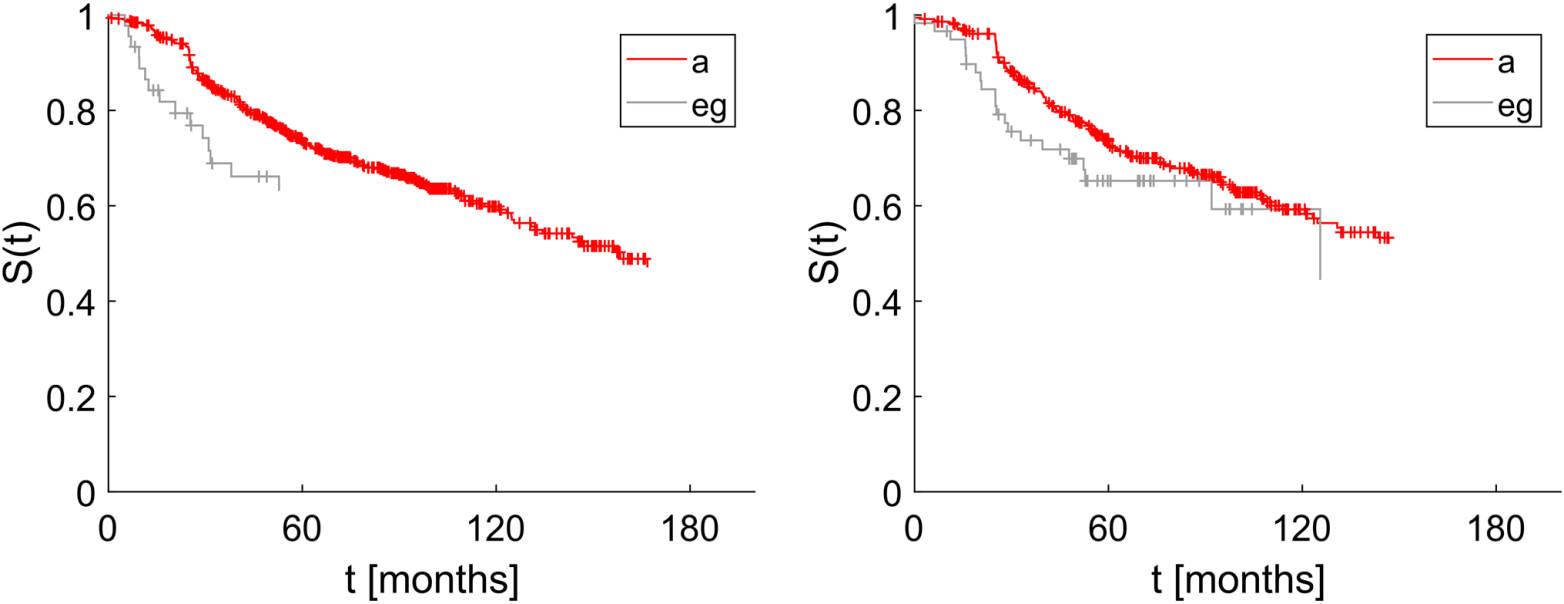
Positive hormone receptor status correctly and erroneously assigned: impact on disease free survival. Left panel: Estrogen receptor status assessed correctly as true positive (label a, 1562 patients in all, 648 of which had survival data) and false positives (label eg, 97 patients in all, 45 of which had survival data), Wilcoxon test *p* = 0.03. Right panel: Progesterone receptor status assessed correctly as true positive (label a, 808 patients in all, 362 of which had survival data) and false positives (label eg, 116 patients in all, 59 of which had survival data), Wilcoxon test *p* = 0.08

Kaplan Meier estimates of disease-free survival were computed separately for positive estrogen receptor status assigned correctly ({ER_a_}) and erroneously ({ER_e,g_}), see Fig. 5, left panel. Please note that survival data do not exist for all patients in our dataset and survival plots are based on a subset of patients within the corresponding flow (a–h).

### Possibly lacking versus unnecessary anti-HER2 therapy

**Fig. 6:**
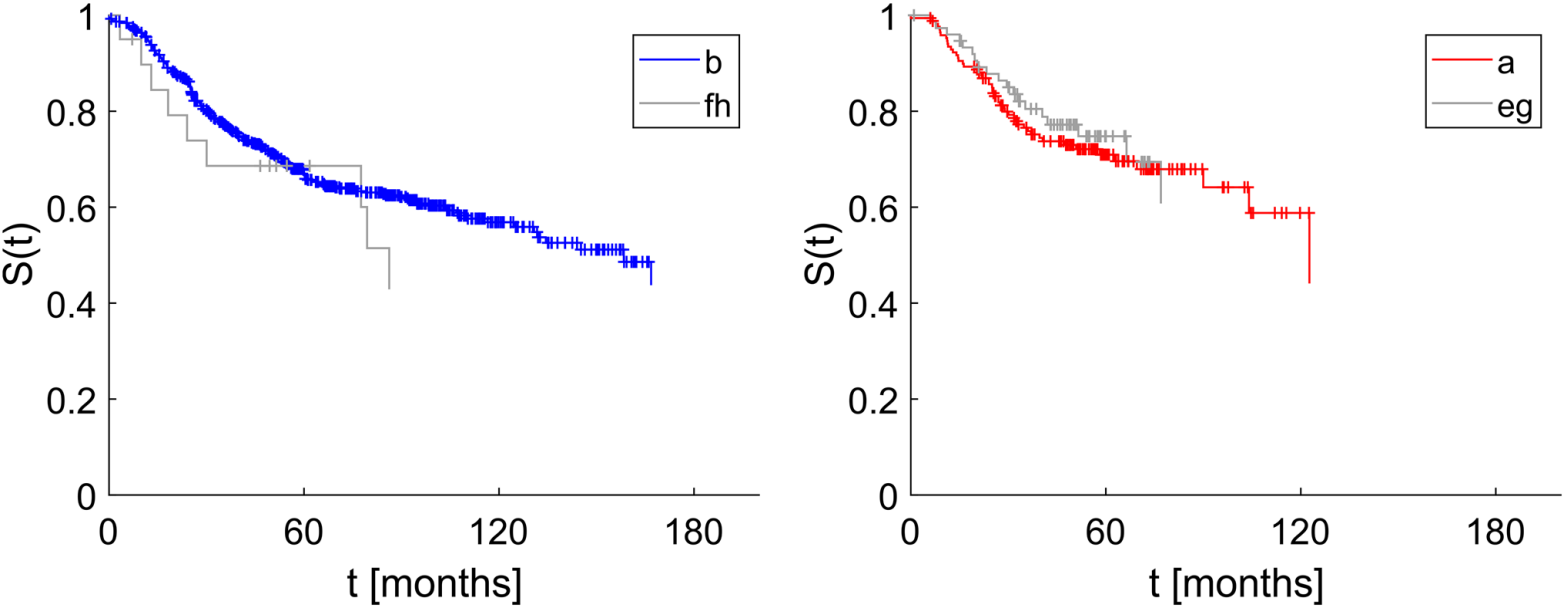
Impact on disease free survival of erroneously assessed HER2 status. Left panel: True negative assessed HER2 (label b) versus false negative (label f, h), Wilcoxon test *p* = 0.41. Note that out of 1772 patients in flow b, survival data were available only for 690 patients. Likewise, out of 33 patients in flows f or h, survival data were available only for 20 patients. Right panel: True positive assessment of HER2 (label a) versus false positive (label e, g), Wilcoxon test *p* = 0.47. Note that out of 458 patients in flow a, survival data were available only for 362 patients. Likewise, out of 181 patients in flows e or g, survival data were available only for 59 patients

In our cohort 1805 patients have been assessed ${\text{HER2}}_{{{\text{IHC}}}}^{ - }$, out of which 1772 were assessed correctly (flow b in Fig. 4, set {HER2_b_}). Only 13 of these have been corrected towards positive (flow f) and 20 rendered inconclusive (flow h). The merged set {HER2_f,h_} = {HER2_f_} ∪ {HER2_h_} is comprised of 33 patients who should have received anti-HER2 therapy, but actually did not. The effect of possibly depriving anti-HER2-therapy is shown in Fig. 6, left panel.

On the contrary, 639 patients have originally been assessed ${\text{HER2}}_{{{\text{IHC}}}}^{+}$, out of which 458 were confirmed, 85 corrected towards negative (flow e) and 96 rendered inconclusive (flow g). The merged set {HER2_e,g_} = {HER2_e_} ∪ {HER2_g_} is comprised of 181 patients who may have received unnecessary anti-HER2 therapy. The impact on disease-free survival is shown in Fig. 6, right panel.

### Enhanced precision of receptor status: impact on outcome

IHC estimates rejected or even corrected by GE & CO definitely represent improvements in diagnostic quality. Corrected cases might receive more adequate therapies (flows e and f). Rejections (flows i and h) may be seen as informative flagging, suggesting to proceed with refined diagnostics prior to a final decision on therapy.

In displaying the impact on outcome, we merge corrections and rejections, e.g. show that the disease free survival for erroneously positive assigned ER (set {ER_e,g_}) is worse than for confirmed positive cases (set {ER_a_}), Wilcoxon test, *p* = 0.03, see left panel Fig. 5.

For PGR, the negative effect of wrong assignments cannot be substantiated (right panel Fig. 5), survival curves fail to show significant differences (Wilcoxon test, *p* = 0.08). The reason may lie in the fact that patients falsely negative in PGR nevertheless received anti-hormone therapy, due to being assessed ${\text{ER}}_{{{\text{IHC}}}}^{+}$.

Please note that the numbers of erroneously assigned receptor status are comparatively low and statistical test results are therefore insignificant in many cases. However, such findings are nevertheless highly important for the patients concerned, and their relevance must not be judged according to *p*-values.

Strictly speaking, the worse survival of patients with ill-assigned IHC-estimates could also have other causes than suboptimal therapy. However, since we know that therapy was likely suboptimal in these cases, it seems the most probable cause and worth being improved.

All in all it is obvious that the number of assignments increases by adding a co-gene.

It is important to understand that this is achieved by the intake of additional information given by the co-gene rather than by relaxing the threshold, *S*_0_, of acceptance. In fact, relaxing the threshold, *S*_0_, would also increase the number of seemingly conclusive assignments—at the cost of concomitantly increasing the rate of wrong assignments, however. Fiddling around with the threshold would only seem to be an improvement. Adding information from a co-gene, however, leads to a real and substantial improvement.

Another issue pertains to the number of co-genes to be considered for each receptor. Of note, adding correlated variables does not confer much additional information. Each variable—considered on its own—holds valuable information, and a statistical test would recommend its inclusion. However, the theory of feature selection recommends caution so as to avoid overfitting due to including a whole bunch of such correlated variables. As broadly described in the literature, many expression profiles up to now have suffered from overfitting, yielding results not reproducible for newly incoming patients.

### Setting the precision threshold

We have chosen the threshold probability, *S*_0_, for acceptance exactly at the logit of precision of a positive IHC measurement without any further information from gene expression.

The reason for this is that any evidence from expression data not contradicting the IHC measurement should yield a definite result.

### Different clinical weights of false positive and false negative assessments

In this work we reveal the impact of erroneously assessed receptor status *on disease free survival* and ignore all other aspects, e.g. side effects and quality of life being reduced by unnecessary treatment.

In an overall optimization one would have to include weights (judged by experts and patients) in order to tune sensitivity versus specificity of all assessments involved in a comprehensive manner. In particular, gains and losses due to falsely positive and negative are often assumed symmetric for simplicity—but this does not sincerely reflect reality.

A detailed analysis of gains versus losses would be needed, as a matter of fact. Gains in lifetime may be weighed against losses in quality of life for each type of correction envisaged (flows e, f, g and h). Should different sets of weights be advocated (e.g. by different panels of doctors and/or patients), slightly different strategies would mathematically result as respective optima. On the contrary, should ethic discussions arise and call for quantitative arguments, this work could readily provide ‘criteria and scores for ethic strategies’ in terms of lifetime.

This work helps to better identify patients for relevant and more appropriate therapy with long overall survival.

## Materials and methods

### Study selection, normalization and co-genes

The dataset for this study has been assembled as follows [25]: out of several hundred breast cancer studies on Gene Expression Omnibus (GEO), which use the Affymetrix chip U133A + 2.0 (‘platform GPL570’ in GEO), we retained only those with 12 samples or more and data for receptor status and/or survival. Out of these 43 studies, 5 were dismissed due to incompatible normalization and two more because of insufficient receptor status. We finally used 36 breast cancer studies from gene-expression omnibus, see Table 9, curated and normalized them as described in Supplementary Materials and Methods.

**Table 9:** List of series-IDs (GSExxxx) and sample-IDs (GSMxxxxx) downloaded from gene expression omnibus (GEO) to be used in the current work. As an example we show the first few IDs out of the first two series. The full list can be downloaded from the Supplementary Table

GSM-ID	GSE-ID	
GSM124996	GSE5460	Series GSE5460
GSM125003	GSE5460	
GSM125005	GSE5460	
GSM125007	GSE5460	
GSM125022	GSE5460	
GSM125023	GSE5460	
GSM125039	GSE5460	
GSM125042	GSE5460	
…	…	
GSM151259	GSE6532	Series GSE6532
GSM151260	GSE6532	
GSM151261	GSE6532	
GSM151262	GSE6532	
GSM151263	GSE6532	
…	…	

**Fig. 7:**
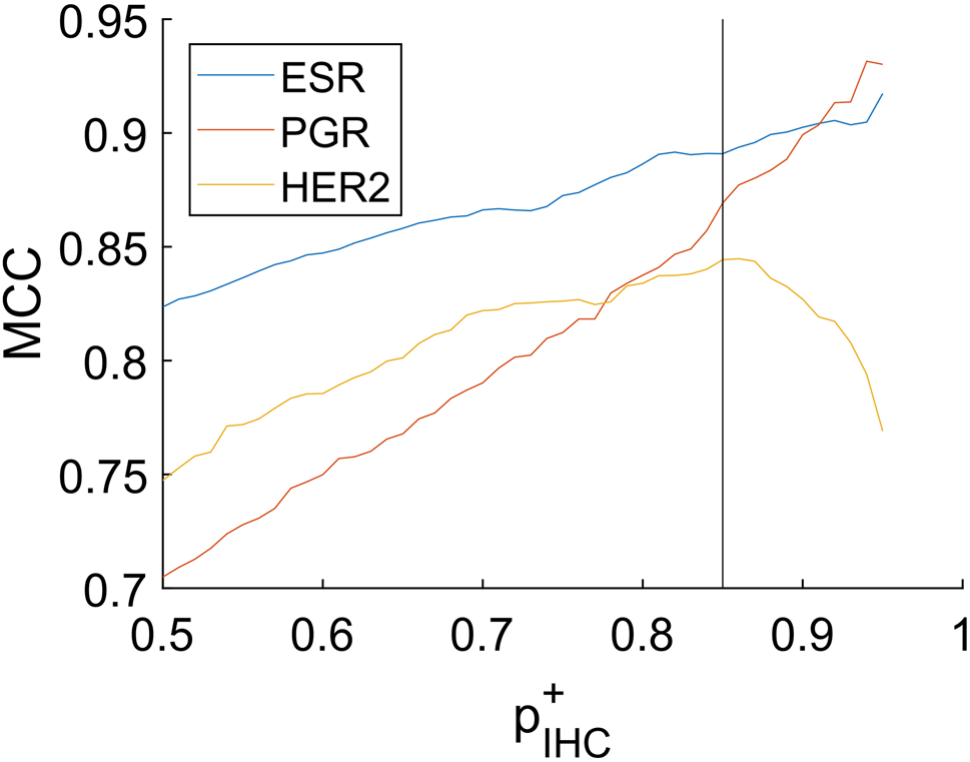
Agreement between IHC and gene-expression measurements. The agreement is measured by the Matthew coefficient [23]. It can be shown [24] that MCC is suitable also for imbalanced group size as in the case of HER2. Setting $p_{{{\text{IHC}}}}^{+}$ entails a certain threshold via ${S_0}\,=\,\log \left( {p_{{{\text{IHC}}}}^{+}/\left( {1 - p_{{{\text{IHC}}}}^{+}} \right)} \right)={\text{logit}}\,p_{{{\text{IHC}}}}^{+}$, the optimum value 0.85 being indicated by the reference line. The higher one chooses $p_{{{\text{IHC}}}}^{+}$, the higher the threshold (*S*_0_) results above which an expression measurement is considered conclusive. Concomitantly, with rising threshold, the agreement between IHC and GE also rises, as reflected by an increasing MCC. Beyond $p_{{{\text{IHC}}}}^{+}=0.90$, however, only few gene-expression measurements remain conclusive, causing the graphs to fluctuate due to sparsity of data. Accordingly, there is no special meaning to the fact that the MCCs for ER and PGR further increase while the MCC for HER2 declines in the rightmost parts

Receptor-genes are uniquely defined for ER, PGR and HER2, and hence their expression values can directly be used. As opposed, possible co-expressed genes have to be selected according to criteria to be defined. To these end we developed and performed a co-expression check, based on intricate criteria, spotting those genes capable to yield maximum information on top of what is known from the very receptor-genes. Finally we end up with AGR3 as co-gene for ESR1, ESR1 as co-gene for PGR and PGAP3 as co-gene for Her2. For details see the Supplementary Materials and Methods and Fig. 7.

### Information extraction and modelling

We performed logistic regressions to model the impact of gene-expression (of genes and co-genes) on receptor status and fused information from three sources (IHC, expression of receptor gene and co-gene) via the product of odds to arrive at a unique and most reliable assessment for each receptor and single patient. For details see the Supplementary Materials and Methods.

### Fusion of information from different sources

Of note, the step-wise increase of information and reliability, as quantified in Table 2, can most vividly be presented in Sankey diagrams, see Figs. 2, 3 4, 8, 9 and 10. They display clearly, how many patients arrive at increasingly secure and precise receptor diagnostics as a result of step-wise fusion of OMICs data (IHC, expression of receptor-genes and expression of co-genes).

**Fig. 8:**
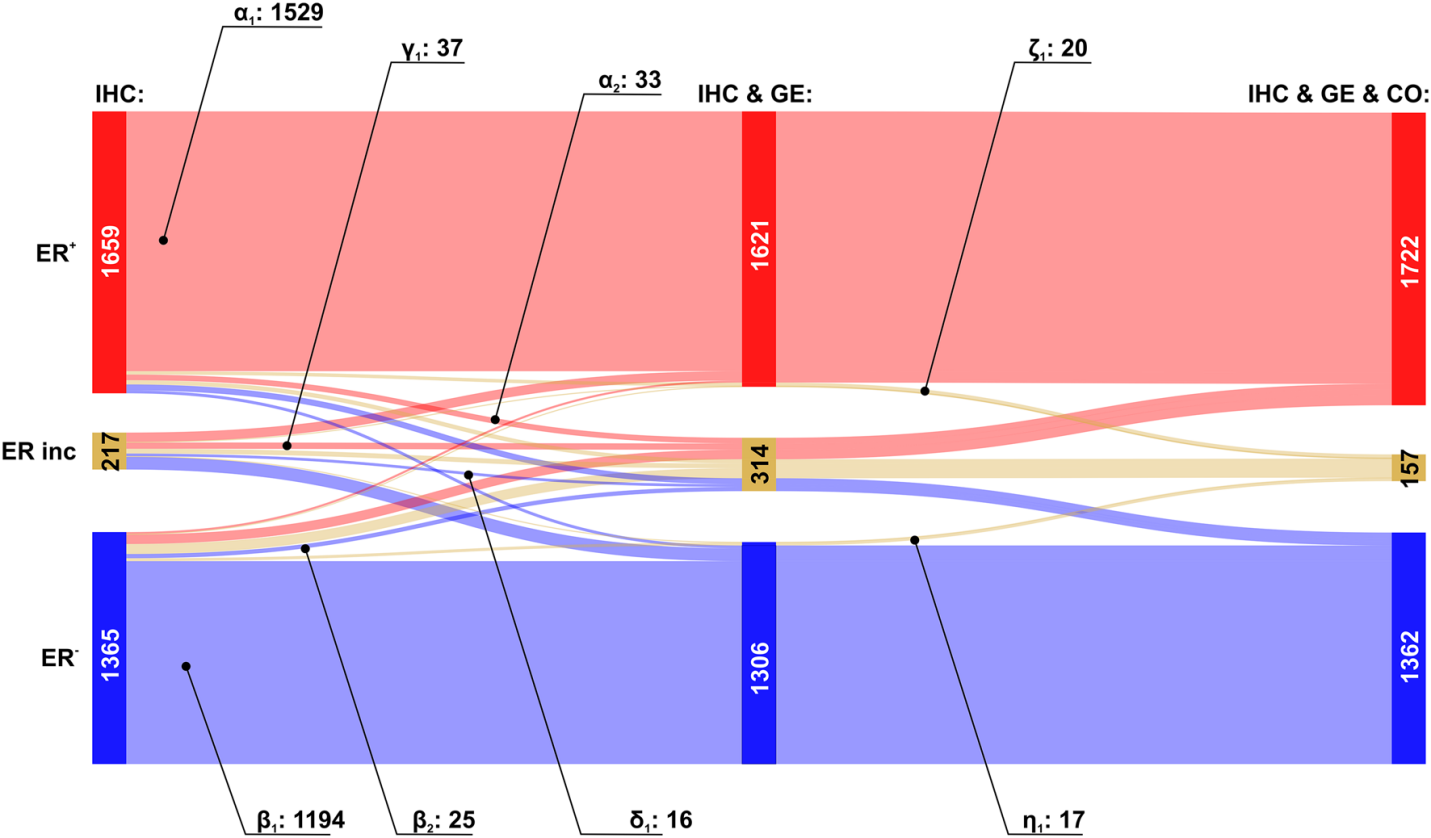
Estrogen receptor diagnosis: patient flows due to adding receptor gene and co-gene. The impact of additionally considering expressions of receptor gene and co-gene is visualized in terms of patient flows (Sankey diagram). As information increases (from left to right) some patients flow between categories. Stripes of flows are coloured according to their final destination, e.g. red, if a patient finally ends up being assessed ER^+^, regardless which category he originated from. Left columns of Sankey diagram: number of patients classified on basis of ‘IHC only’ (red: ER^+^, beige: ER^inc^, blue: ER^−^). Middle columns: number of patients in above groups after adding information from gene-expression (GE) of receptor gene *ESR1* (classification according to ‘IHC & GE’). Right columns of Sankey diagram: numbers of patients after adding information from co-gene expression (CO) of co-gene *AGR3* (classification according to ‘IHC & GE & CO’)

**Fig. 9:**
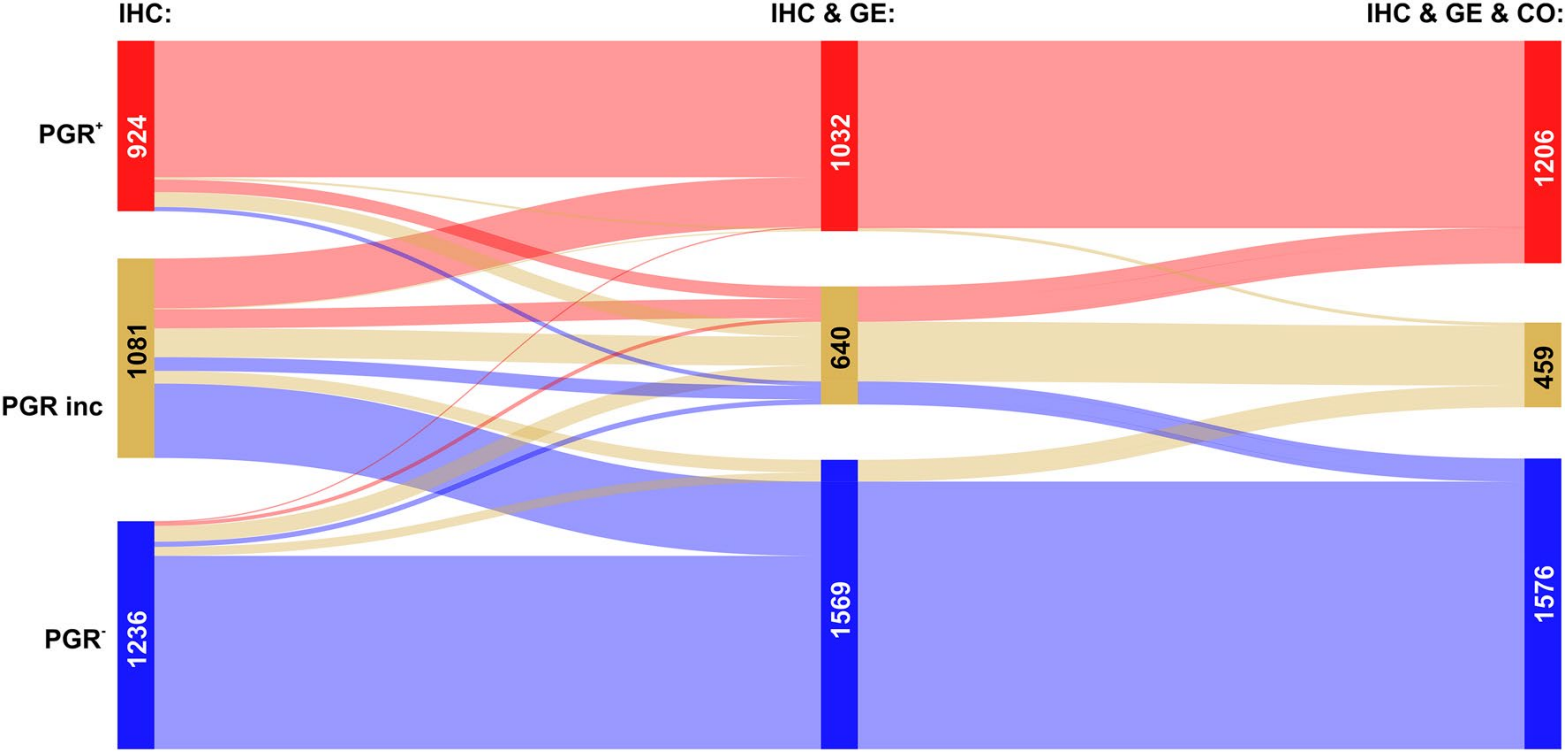
Progesterone receptor diagnosis: patient flows due to additionally considering expression of receptor gene and co-gene. Left column of Sankey diagram: number of patients classified (red: PGR^+^, beige: PGR^inc^, blue: PGR^−^) on basis of IHC. Middle column: Number of patients in above groups after adding information from gene-expression (GE) of receptor gene *PGR*. Right column of Sankey diagram: number of patients classified when additionally the co-gene *ESR1* is considered

**Fig. 10:**
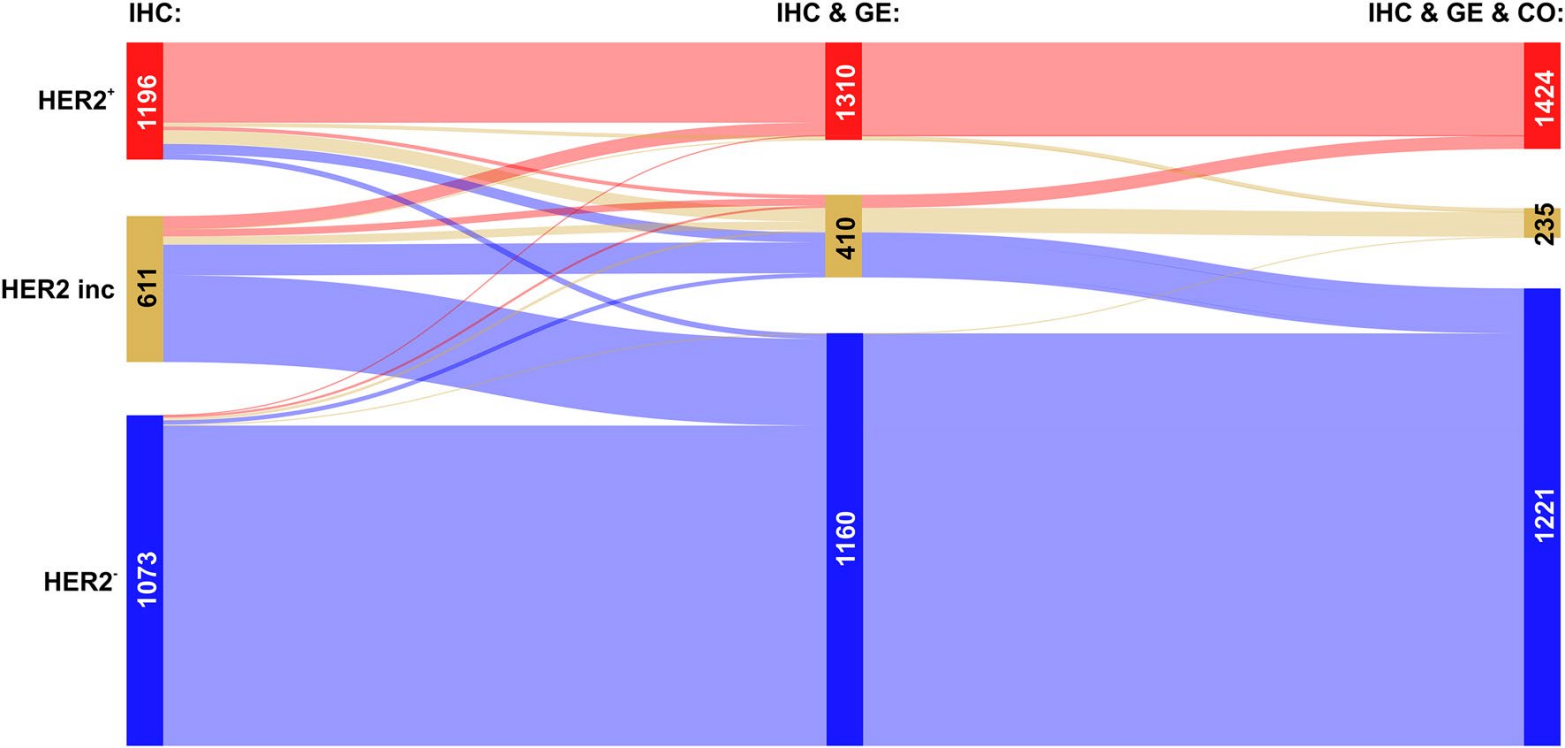
HER2 diagnosis: Patient flows due to additionally considering expression of receptor gene and co-gene. Left column of Sankey diagram: number of patients classified (red: HER2^+^, beige: HER2^inc^, blue: HER2^−^) on basis of IHC. Middle column: number of patients in above groups after adding information from gene-expression (GE) of receptor gene *ERBB2*. Right column of Sankey diagram: number of patients classified when additionally the co-gene *PAGP3* is considered

## Electronic supplementary material

Below is the link to the electronic supplementary material.

Supplementary material 1 (DOCX 110 KB)

Sankey diagrams with interactive capability are available for detailed reference to numbers of patients in flows. Supplementary material 2 (XLS 225 KB)

## Footnotes

1 Precision is also called ‘positive predictive value’ according to the terminology of machine learning.
