# Supplementary material for: Co-expressed genes enhance precision of receptor status identification in breast cancer patients
Source: Breast Cancer Res Treat. 2018 Aug 16;172(2):313–26. doi: 10.1007/s10549-018-4920-x (PMC6208909; doi:10.1007/s10549-018-4920-x)
Supplement: Supplementary file 1 — Supplementary material 1 (DOCX 110 KB) [file 10549_2018_4920_MOESM1_ESM.docx]

# Supplementary Materials and Methods

## Gene expression data and normalization

We used data from gene expression omnibus as described previously [1], comprised of *N*_sample_ = 3241 individual gene expression samples (Affymetrix U133A+2.0 arrays) out of 36 studies on breast cancer, also containing receptor data. A list of GSE- and GSM-numbers of the samples finally used is given in Table 9.

Normalization was performed on probes with the GC Robust Multi-array Average (GCRMA) procedure [2, 3], using the affygcrma function of the Matlab Bioinformatics Toolbox with default parameters. This results in expression values for 54675 probe sets for each of the 3241 samples.

Note that we consider the (computationally intensive) GCRMA normalization as the best method currently available. It is superior in accuracy to the older and commonly used RMA normalization in particular when dealing with probe sets showing small variances, making background noise an issue. However, since the probe sets used in the present work exhibit large variances, even RMA would yield similar results.

For our model, only 6 probe sets are required, three for the receptor genes and three for the co-genes.

## Co-expression check: Selecting co-expressed genes

To further enhance the reliability of receptor status estimation we additionally consider coordinately expressed genes (CO). Several candidates for receptor co-genes are mentioned in the literature [4, 5]. We looked for co-genes numerically as follows (‘co-expression check’): The co-gene must not be the receptor gene itself but nevertheless discriminate receptor status as accurately as possible, compared against IHC-data (gold standard).

We applied the limma-package [6] to CEL-files comparing IHC receptor positive against negative. Mapping probe sets to genes finally yields a list of putative co-genes, sorted by descending *t*-values, see Table 5, Table 6 and Table 7.

For the ER, the very receptor gene (*ESR1*) scores top, as expected, and we obtain *AGR3* as second, i.e. as the top scoring co-gene.

For the PGR, the very receptor gene, *PGR*, scores top, as expected. Second, however, scores *ESR1*, the receptor gene for estrogen. Hence *ESR1* plays a double role, as receptor gene and as co-gene.

Likewise, Table 7 shows genes and prob sets discriminating for HER2. Surprisingly, the very receptor gene, *ERBB2*, does not score top but comes second. Yet we consider it the receptor gene. Top scoring is *PGAP3*, which we hence select as co-gene.

We finally selected: *AGR3* as co-gene for *ESR1*, *ESR1* as co-gene for PGR and *PGAP3* as co-gene for HER2, see also Table 1 and Table 8.

## Why to perform logistic regression for genes and co-genes

In our previous paper [1] we used the expectation maximization algorithm, see p. 275 in [7], to model gene expression. Since we considered receptor genes only, the two peaks in such an expression profile clearly corresponded to the two receptor status – one peak to receptor negative, the other peak to receptor positive. The expectation algorithm, being an unsupervised method, retrieved this correspondence and also offered the advantage of not drawing on IHC-information, i.e. it operated independently of any information to be predicted.

When searching for a putative co-gene (besides the very receptor gene), the situation is different. The expression profile of any arbitrary gene might well exhibit two peaks, but there is no guarantee that these peaks actually correspond to those two receptor status in question. Two peaks might as well result from any other confounding variables, assuming two possible values. To ensure that receptor status is the very target being modelled, we prefer a supervised method such as logistic regression. The generated model is then a logit function (rather than a responsibility function), and its output for a given patient is a probability value (for being receptor positive), see Fig 1.

## Results and quality of receptor prediction from logistic regression

Performing a logistic regression for IHC estimates versus gene-expression, $x_{\text{GE}}$, yields a predicted probability $p_{\text{ER}_{\text{GE}}^{\text{+}}}(x_{\text{GE}})$ for receptor positivity:

|  | $p_{\text{ER}_{\text{GE}}^{\text{+}}}(x_{\text{GE}})=\frac{1}{1+e^{\beta_{0}^{\text{GE}}+\beta_{1}^{\text{GE}}x_{\text{GE}}}}$ | (1) |
| --- | --- | --- |

The regression coefficients for each of the receptor genes and co-genes are given in Table 8.

The number of samples (last column) results from the number of IHC estimates available, upon which logistic regression is based.

The quality of this logistic regression can be quantified in various ways. We have chosen two commonly used parameters, the Area Under the Curve (AUC) of the Receiver Operating Characteristic (ROC) and the deviance of fit^[[1]](#footnote-1)^ (DoF), see e.g. p. 118 in [8].

In terms of AUC, Table 8 shows a slight superiority of the genes versus the co-genes for ER and PGR while the co-gene *PGAP3* performs the same as HER2.

Another measure of regression quality is the deviance of fit [8], DoF, see last but one column in Table 8. Larger DoF indicates more deviation (less quality). Again, receptor genes outperform co-genes for ER and PGR but not for HER2. When comparing DoF between different receptors (e.g. ER vs PGR) one must bear in mind that the DoF increases with the number of samples (last column).

## Joining information from different sources

### Concise decision formula

For information joining, we convert precision, *p,* into odds, $\psi=\frac{p}{1-p}$, an then aggregate odds by multiplication:

|  | $\psi^{+}=\psi_{\text{IHC}}^{+}\cdot\psi_{\text{GE}}^{+}\cdot\psi_{\text{CO}}^{+}$ | (2) |
| --- | --- | --- |

Taking the logarithm of aggregated odds yields a simple sum, lending itself as a profound and very natural score

|  | $S^{+}=\log(\psi_{\text{IHC}}^{+}\cdot\psi_{\text{GE}}^{+}\cdot\psi_{\text{CO}}^{+})=\log(\psi_{\text{IHC}}^{+})+\log(\psi_{\text{GE}}^{+})+\log(\psi_{\text{CO}}^{+})$ | (3) |
| --- | --- | --- |

Inserting the constant precision for IHC and the logit representation for gene expression yields:

|  | $S^{+}=\log\left(\frac{p_{\text{IHC}}^{+}}{1-p_{\text{IHC}}^{+}}\right)-\left(\beta_{0}^{\text{GE}}+\beta_{1}^{\text{GE}}x_{\text{GE}}\right)-\left(\beta_{0}^{\text{CO}}+\beta_{1}^{\text{CO}}x_{\text{GE}}\right)$ | (4) |
| --- | --- | --- |

*S*^+^ is computed for each patient and then checked against a threshold, *S*_0_. For setting the magnitude of *S*_0_, see the section below. If $S^{+}>S_{0}$, the patient is considered receptor positive, if $S^{+}<-S_{0}$ receptor negative, and for intermediate values as inconclusive. This rule may be packed into a concise mathematical formula

|  |  | (5) |
| --- | --- | --- |

with I() being the indicator function^[[2]](#footnote-2)^. Equation (5) has been designed to yield receptor status results as $K=1$ for positive, $K=-1$ for negative, and $K=0$ for inconclusive receptor status.

### Multiplying odds

In our previous work we have considered only two sources of information (IHC and GE). Any contradictions between these had to be considered an ‘inconclusive’ receptor status. In other words: To be on the safe side, only those evidences which agreed were accredited as true.

Now we consider three sources of information (IHC, GE, CO), which makes the situation more promising but also more complicated. Two of three sources might agree and one may contradict the findings. To reach a decision in such a situation, one could draw on some weighting (scoring) system. Such a system, however, would have to be postulated somewhat arbitrarily, from which we refrain and rather switch to another paradigm for joining information.

We proceed to odds, providing an elegant concept for characterizing the output from each source of information. Several odds may then be multiplied to yield overall odds. Only after this final step of multiplication (and not before), the overall odds may - in some cases - be classified ‘inconclusive’ (see equ. (5)).

We refrain from considering the category ‘inconclusive’ separately for each source of information but rather categorize the final probabilistic outcome. This renders unnecessary any arbitrary weighting of information sources against each other.

## Setting the threshold

The threshold, *S*_0_, is set in a way that no definite conclusion can be drawn from IHC alone. In cases where gene expression does not contribute any affirmation, the decision about receptor status should render ‘inconclusive’.

Implementing this paradigm we set this threshold *S*_0_ equal to the logit of precision. Based on the assignment $p_{\text{IHC}}^{+}=0.85$ (and hence $p_{\text{IHC}}^{-}=0.15$) we obtain $S_{0}=\log\left(0.85/0.15\right)\approx1.735$, see Equ. (2).

Setting different thresholds, *S*_0_, changes the rate to which IHC is in agreement with GE, as quantified by the Matthew-Correlation Coefficient [9], see Fig 7.

The paradigm itself as well as the consequences of larger and smaller choices of *S*_0_ have been scrutinized in the discussion.

## Three steps of estrogen receptor diagnostics improvement traced in Sankey diagrams

Table 2 shows overall numbers of patients in respective categories of diagnosed receptor status (+, inc, -). These numbers do not reveal, however, how many patients changed from one category to another as a result of additionally taking GE and CO into consideration.

To uncover these flows of individual patients between categories, we first note that the scoring formulae, see main article, equations 2 and 3, have been designed to be readily expandable or collapsible for dealing with more or fewer sources of information, respectively. For example, if we would consider just IHC and GE, the last additive term would not appear, and equation 2 (main article) would read

|  | $S^{+}=\log\left(\frac{p_{\text{IHC}}^{+}}{1-p_{\text{IHC}}^{+}}\right)-\left(\beta_{0}^{\text{GE}}+\beta_{1}^{\text{GE}}x_{\text{GE}}\right)$ | (6) |
| --- | --- | --- |

For this scenario, the resulting numbers of patients are listed in the columns headed ‘IHC & GE’ of Table 2. As a consequence, the number of inconclusive cases is reduced.

We have evaluated, e.g., how many patients were at first diagnosed receptor positive by ‘IHC alone’ and then changed to negative after being diagnosed by ‘IHC & GE’, etc. In other words, enriching the method ‘IHC’ by ‘gene expression’ (GE) yields several flows of patients, shown in the left-to-middle parts of Fig 8, Fig 9 and Fig 10.

Likewise, further addition of co-expression on top of GE, yields the improvements shown in the middle-to-right parts of these figures.

Having introduced the Sankey diagrams within diagnostic refinement we illustrate and comment on several key features. Please note that Sankey diagrams are also available under additional material and there they offer interactive features to display numbers of patients in particular flows.

The Sankey diagram displays changes in estimated receptor status in steps with increasing information: in a first step due to adding GE-information, and in a second step due to adding CO-information.

Since we discriminate three different categories (+, -, inconclusive), each step of adding information may shift a patient in any of the three different categories (+, -, inconclusive). In Table 3 (in the result section) we have only considered the initial (IHC) and final information (IHC & GE & CO). Between these states of information 3 x 3 = 9 flows between categories may occur.

In this more detailed section we also consider the intermediate step of adding just GE, already giving rise to 9 flows. The next step of adding CO generates another 9 possible flows, thus generating 27 possible flows from an initial IHC estimate towards the final result based on all information available. In the following we shall exemplify some of them.

It is not surprising that the flows from an initial IHC estimate towards an unchanged final result are populated highest. For ER^+^, most patients (n = 1529) are first confirmed by GE and a second time by CO (flow α_1_). Few patients (n = 33) are first rejected by GE but then re-assigned (i.e. confirmed) ER^+^ by CO (flow α_2_). Likewise for ER^-^, most patients (n = 1194) are first confirmed by GE and a second time by CO (flow β_1_). Few patients (n = 25) are first rejected by GE but then re-assigned (i.e. confirmed) ER^-^ by CO (flow β_2_).

However, most interesting are flows originating in a missing IHC estimate, then still being inconclusive after adding information from GE, but finally rendered conclusive (yielding a definite result) after adding information from CO. One example is a group of 37 patients originating as inconclusive from IHC, still inconclusive after adding GE (flow labelled γ_1_ in Fig 8), and finally allocated as ER^+^ after adding CO:

Likewise, 16 patients originally IHC inconclusive remain so after adding GE (flow labelled δ_1_) but are finally allocated towards a definite status ER^-^:

Such cases represent a significant diagnostic improvement, due to adding CO information.

Also of interest are cases which have been assigned a definite status after adding GE but are questioned (i.e. rejected) upon additionally considering CO and finally end-up in category inconclusive. Examples are the flow of 20 patients labelled ζ_1_, see Fig 8,

and the flow of 17 patients labelled η_1_:

These cases also represent improvements with respect to our previous paper [1] in which we considered additional information only from GE: If a receptor status results inconclusive under full information and has to be re-assessed, a possibly suboptimal treatment may be avoided.

## Sankey diagrams reveal intricate features of stepwise diagnostics

Taking the 2 steps of ER diagnostics (see Fig 8) several benefits of enriching information as proposed can be delineated:

1. The overall number of inconclusive cases shows intricate changes with each piece of information added (217 → 314 → 157).
   1. After adding information from GE, about one quarter of patients lacking IHC^[[3]](#footnote-3)^ estimates (55 out of 217) flow towards ER^+^ and another third towards ER^-^ (75 out of 217), and patients in both groups stay there.
   2. In 6 cases adding GE information first renders a definite decision – which is questioned, however,- after adding CO.
   3. From those inconclusive under ‘IHC & GE’ (n = 314) about one third (n = 126) flows to ER^+^, another quarter (n = 76) to ER^-^ and the rest remains inconclusive.
   4. In 28 cases neither adding Ge information nor adding CO renders a secure decision.
2. Nevertheless, some cases will shift from decidable (ER^+^ or ER^-^) to inconclusive due to added information. To be precise, they were just *seemingly* decidable based on previous information. Adding information added doubt in their initial assessment, and we know that such doubt is legitimate, given the rate of erroneous IHC- estimates [10, 11]. If these patients can be flagged to be re-assessed [12], more adequate treatments could result and we may well speak of a ‘benefit from doubt’.

Out of those assessed ER^+^ according to IHC, a considerable number is re-diverted towards ‘inconclusive’ (n = 93) when considering ‘IHC & GE’. Some of them (n = 33) return to ER^+^ due to additional evidence from CO, others remain inconclusive (n = 25) and some even flow to ER^-^ (n = 35).

1. Very few patients assessed ER^+^ according to IHC may even end up as ER^-^ (n = 52), see the blue flows originating from ER^+^ at the very left of the plot. This may occur already after adding GE or in the following step, after adding CO.
2. In general we observe that the second piece of information (from GE) causes larger flows between categories than the third piece (CO) does:
   1. Adding CO was never seen capable to invert a decision inferred from ‘IHC & GE’. Its major significance lies in clarifying some inconclusive cases towards ER^+^ (n = 126) and towards ER^-^ (n = 76).
   2. Cases definitely ER^+^ under IHC & GE are rarely rendered inconclusive after adding CO (n = 25).
   3. Likewise cases ER^-^ under IHC & GE are rarely rendered inconclusive after adding CO (n = 20).

## Three steps of PGR and HER2 diagnostics

Enriching diagnostic information for PGR and HER2 proceeds exactly along the same routes as for ER. We display the Sankey diagrams (Fig 9 and Fig 10) and give short descriptions.

When compared with ER, more IHC estimates are missing in our data for PGR, see Fig 9. Consequently, already the first step of adding information (from GE) renders numerous patients definite (positive as well as negative) which are confirmed later on by CO (assignments).

Again, corrections are small in number.

Regarding ER and PGR, almost equal numbers are observed receptor positive and negative. Conversely, the prevalence is drastically skewed for HER2: About 90% of patients are HER2 negative and only 10% positive. Accordingly, cases allocated by GE & CO split according to the same ratio, see Fig 10.

## Discussion of methodological improvements

## Obtaining additional information on receptor status from gene expression may proceed along several concepts. Multi-gene signatures have been trained via linear discriminant analysis or decisions may be obtained from multiple single-gene- regressions [13] and then balloted. Or else, information from different sources may be joined via a scoring system, as we demonstrated in our previous paper [1].

Our approach given in equation 2 (main article) suggests to add co-genes in order to increase robustness. Usually, adding prognostic variables implies some risk of overfitting. In our case, however, this risk is mitigated by the flatness of logistic regression curves for co-genes with larger DoF. However, observed improvements in prediction were never larger than the uncertainty (≈ 10%) of our underlying training data.

## In the present work we aim at avoiding the setting of thresholds for each gene and also a subsequent, somewhat arbitrary scoring of the joint result. At the same time we try to do without declaring single sources of information as inconclusive if their numerical estimates should lie within respective, critical regions.

## As a solution, we joined information from different sources (IHC, GE and CO) by first computing odds and then multiplying them. It is only in the very last step of this procedure that a joint estimate may be declared ‘inconclusive’, and only one threshold has to be set for this purpose (instead of many). We think this represents a significant methodological improvement.

Please note that multiplication of odds is commutative (the order may be interchanged without changing the result) and renders irrelevant which of the information (GE, CO) should have priority, instead they contribute equally. Should this be unwanted, weights may be introduced in a most transparent way.

We introduced the use of Sankey diagram (Fig 8) to demonstrate several interesting consequences of stepwise enriching information.

The precision of the method we have proposed may be estimated from the fact that 18 different flows actually occur, out of 27 possible, all in all.

# References

1. Kenn M, Schlangen K, Cacsire Castillo-Tong D, Singer CF, Cibena M, Koelbl H et al (2017) Gene expression information improves reliability of receptor status in breast cancer patients. Oncotarget; 8:77341-77359.

2. Wu Z, Irizarry RA, Gentleman R, Martinez-Murillo F, Spencer F (2004) A Model-Based Background Adjustment for Oligonucleotide Expression Arrays. Journal of the American Statistical Association; 99:909-917.

3. Wu Z, Irizarry RA (2004) Preprocessing of oligonucleotide array data. Nat Biotechnol; 22:656-658.

4. Ikeda K, Horie-Inoue K, Inoue S (2015) Identification of estrogen-responsive genes based on the DNA binding properties of estrogen receptors using high-throughput sequencing technology. Acta Pharmacologica Sinica; 36:24-31.

5. Lin CY, Ström A, Vega VB, Li Kong S, Li Yeo A, Thomsen JS et al (2004) Discovery of estrogen receptor α target genes and response elements in breast tumor cells. Genome Biology; 5:R66.

6. Ritchie ME, Phipson B, Wu D, Hu Y, Law CW, Shi W et al (2015) limma powers differential expression analyses for RNA-sequencing and microarray studies. Nucleic Acids Res; 43:e47.

7. Hastie T, Tibshirani R, Friedman J (2009)The Elements of Statistical Learning: Data Mining, Inference, and Prediction*,* edn. 2, Springer.

8. McCullagh P, Nelder JA: Generalized Linear Models**.** edition Second Edition. *Monographs on Statistics and Applied Probability*. London, New York: Chapman & Hall/CRC; 1989.

9. Powers DM (2011) Evaluation: from Precision, Recall and F-measure to ROC, Informedness, Markedness and Correlation. 2 edition;37-63.

10. Laas E, Mallon P, Duhoux FP, Hamidouche A, Rouzier R, Reyal F (2016) Low concordance between gene expression signatures in ER positive HER2 negative breast carcinoma could impair their clinical application. PLoS ONE; 11:e0148957.

11. Wells CA, Sloane JP, Coleman D, Munt C, Amendoeira I, Apostolikas N et al (2004) Consistency of staining and reporting of oestrogen receptor immunocytochemistry within the European Union - An inter-laboratory study. Virchows Arch; 445:119-128.

12. Hammond ME, Hayes DF, Wolff AC, Mangu PB, Temin S (2010) American Society of Clinical Oncology/College of American Pathologists Guideline Recommendations for Immunohistochemical Testing of Estrogen and Progesterone Receptors in Breast Cancer. JOP; 6:195-197.

13. Li Q, Eklund AC, Juul N, Haibe-Kains B, Workman CT, Richardson AL et al (2010) Minimising immunohistochemical false negative ER classification using a complementary 23 gene expression signature of ER status. PLoS ONE; 5:e15031.

14. Boughorbel S, Jarray F, El-Anbari M (2017) Optimal classifier for imbalanced data using Matthews Correlation Coefficient metric. PLoS ONE; 12:e0177678.

1. DoF for a multinomial logistic regression is defined as twice the difference between the maximum achievable log likelihood and that attained under the fitted model. [↑](#footnote-ref-1)
2. The indicator function yields value one if the argument is true, zero otherwise. [↑](#footnote-ref-2)
3. Lacking IHC estimates are labelled ‘undecidable’ in the graphical display in order to be consistent with the terminology used for categories of IHC & GE and IHC & GE & CO, respectively. [↑](#footnote-ref-3)
